# Active Switching of Orbital Angular Momentum of Light Using Metasurfaces Incorporating Vanadium Dioxide

**DOI:** 10.1002/nap2.70040

**Published:** 2026-02-26

**Authors:** Qinghong Lyu, Qiuchen Yan, Yulan Fu, Xiaoyong Hu, Qihuang Gong

**Affiliations:** ^1^ State Key Laboratory for Mesoscopic Physics & Department of Physics, Collaborative Innovation Center of Quantum Matter & Frontiers Science Center for Nano‐optoelectronics Peking University Beijing China; ^2^ School of Physics and Optoelectronic Engineering Institute of Information Photonics Technology Beijing University of Technology Beijing China; ^3^ Key Laboratory for Advanced Optoelectronic Integrated Chips of Jiangsu Province Peking University Yangtze Delta Institute of Optoelectronics Nantong Jiangsu China; ^4^ Collaborative Innovation Center of Extreme Optics Shanxi University Taiyuan Shanxi China; ^5^ Hefei National Laboratory Hefei China

**Keywords:** metasurface, OAM switching, vanadium dioxide

## Abstract

The growing demand for orbital angular momentum (OAM) in optical communications calls for compact efficient beam‐control platforms. Metasurfaces have emerged as a powerful tool for generating OAM beams, yet most designs remain static. Vanadium dioxide (VO_2_), a phase‐change material, with its reversible insulator‐to‐metal transition offers a promising path to dynamic control. However, in existing VO_2_ integrated metasurfaces, its potential is underexploited. It is often used as a thin film that fails to exploit the full refractive index variance. Although recent attempts have incorporated VO_2_ into nanostructures, they remain limited to binary‐phase modulation or involve fabrication‐complex structures that are unsuitable for applications in the telecommunication waveband. Here, we propose a reflective active metasurface at 1500 nm that integrates VO_2_ into metal‐insulator‐metal meta‐atoms. Our design strategy selects the geometry of each meta‐atom based on the phase difference across VO_2_ transition, and its orientation is governed by the proposed equations. Our design enables efficient OAM switching with large topological charge leaps. We numerically demonstrate three metasurfaces that switch OAM states between ℓ = (−1, −3), *l* = (−2, −3), and (4, −1). Our work establishes a versatile and fabrication‐friendly platform for active OAM manipulation, promising advanced applications in high‐capacity optical communications.

## Graphical abstract

This work presents a novel design strategy for active metasurfaces that actively switches OAM modes. The metasurface integrates VO_2_ within MIM meta‐atoms. Operating at 1500 nm, the designed platform enables large leaps in topological charge. For instance, the reflected beam can be switched from a topological charge of −1 in the insulating (M1) phase to −3 in metallic (R) phase.

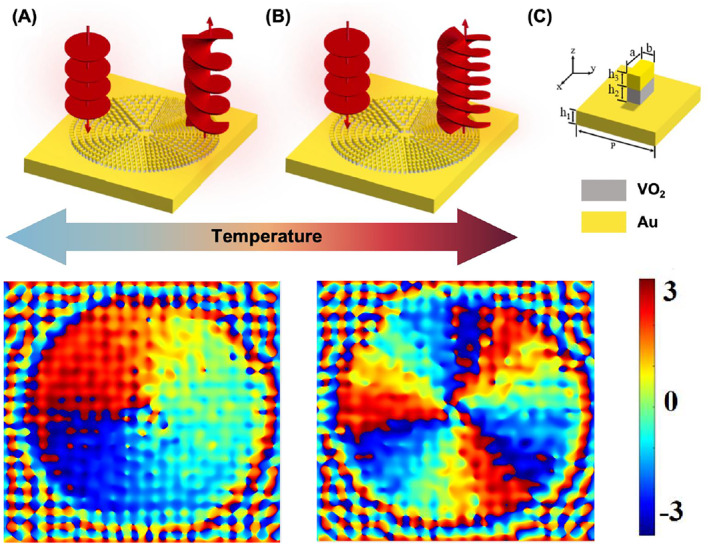

## 1 Introduction

Light, a form of electromagnetic wave that propagates in space and oscillates in time, has been demonstrated to carry spin angular momentum (SAM) and orbital angular momentum (OAM) [1, 2, 3]. Although SAM of light is intrinsically associated with circular polarized (CP) light and only possesses two values of ±ℏ [4] (ℏ is the reduced Planck constant), OAM of light is unbounded (±lℏ) and is characterized by a helical phase distribution exp(ilφ), in which φ is the azimuthal angle and l is the topological charge (TC) [3]. As a new degree of freedom with great potential, OAM modes can be used to increase the optical communication capacity [5, 6] as well as super‐resolution imaging [7] and micro‐manipulation [8], thus making the generation of OAM beams a vital work. Various methods have been developed to generate OAM beams, including forked gratings [9], spiral phase plates [10], q‐plates [11], laser mode conversion [12], spatial light modulators [13], and metasurfaces. Among them, metasurfaces are constituted by subwavelength optical resonator arrays, capable of tailoring the amplitude, polarization, and phase of light [13, 14, 15, 16, 17], providing a new perspective for the generation and manipulation of OAM beams [14, 18, 19, 20].

Recent progress in metasurface design has led to highly efficient and broadband OAM generators. For example, catenary‐based metasurfaces have been demonstrated to achieve continuous and achromatic phase modulation, enabling the generation of perfect OAM beams across a wide spectrum [21]. Such static designs excel in efficiency and bandwidth but lack tunability after fabrication. In parallel, detection and sorting of OAM modes have been addressed via specialized metasurfaces capable of distinguishing both SAM and OAM states [22] as well as through robust measurement schemes for partially coherent vortex beams under perturbations [23]. These works collectively address the generation and sorting of OAM modes, yet they also operate within a static or passive framework.

To fully harness the infinite OAM modes in dynamic systems, such as reconfigurable optical communications [24, 25], active control over the TC is essential. Active metasurfaces have thus been developed to induce dynamic tunability [26, 27, 28], using liquid crystals [29, 30, 31], graphene [32, 33, 34], and phase‐change materials (PCMs) [35].

The optical properties of the PCMs, such as VO_2_, Ge_2_Sb_2_Te_5_ (GST), and GSST [36, 37, 38, 39], change obviously after the phase transition, which brings additional degree of freedom to the design of metasurfaces. Among diverse PCMs, VO_2_ is widely used owing to its mild phase transition temperature *T*. Below temperature *T*, which is 68°C, VO_2_ is an insulator with a monoclinic (M1) structure, whereas above *T*, it is metallic with a rutile (R) structure. Such transition, also known as insulator‐metal transition (IMT), can be stimulated by thermal heating [40], electric current [41, 42], strain [43], and ultrafast pulsed laser [44, 45, 46, 47]. Notably, the ultrafast pulsed laser induced IMT of VO_2_ stands out for its ultrafast switching dynamics, a key advantage for high‐speed photonic applications. Extensive studies have validated its rapid response, for instance, few‐femtosecond extreme UV transient absorption spectroscopy measurements revealed that the phase transition completes within 26 ± 6 fs [46]. Such phase change material has been widely used in active metasurfaces to realize active polarization as well as amplitude and phase control [42, 48, 49, 50, 51, 52, 53].

However, in most active metasurface designs, VO_2_ is utilized merely as a thin layer, not fully leveraging the substantial refractive index contrast between its insulating (M1) and metallic (R) phases. As a result, the potential of VO_2_ is underutilized. Recent advances have sought to overcome this by patterning VO_2_ into nanostructures. For instance, some works achieve dynamic switching by employing complex bilayer architectures where the phase transition switches the dominant response between separate VO_2_ and metal structures [54, 55, 56, 57]. Others have demonstrated functionalities such as hologram switching or polarization control using patterned VO_2_ [58, 59]. However, these approaches often face limitations: they are predominantly confined to the terahertz regime, their multilayer or intricate designs pose significant fabrication challenges for infrared wavelengths. In addition, the tuning mechanism reported in [58] offers only binary phase modulation, which is not conducive to efficient and arbitrary OAM state switching in the optical communication band. Consequently, achieving flexible OAM switching via a simple, fabrication‐friendly, and optically efficient VO_2_‐integrated platform remains a critical and promising task.

**FIGURE 1:**
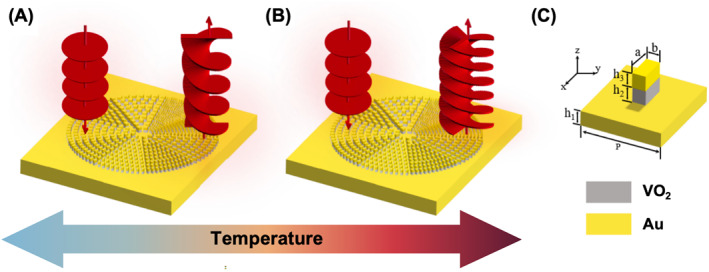
The active switching of OAM via VO_2_ metasurface and the MIM design of meta‐atom. (A) The OAM generation with topological charge of −2 within M1 phase below 68°C. (B) The OAM generation with topological charge of −3 within R phase above 68°C. (C) The schematic of meta‐atom with MIM configuration. The Period of the unit‐cell P is 1.4 times the length of the structural diagonal. The thickness of the Au topping, the VO_2_ layer, and the Au substrate, which is h3, h2, and h1, is 100 nm, 100 nm, and 2 μm.

In the proposed scheme, VO_2_ is incorporated as a part of the metal‐insulator‐metal (MIM) type meta‐atom and the active control of OAM with different TC is achieved, the schematic diagram is shown in Figure 1. By combining the Pancharatnam–Berry (PB) phase and propagation phase of the hybrid meta‐atom, and by further considering the periodicity of phase, we put forward a novel design strategy, in which meta‐atoms are selected based on the phase difference during the VO_2_ transition and oriented via proposed equations. We numerically designed the active metasurfaces at the telecommunication waveband (1500 nm) in reflective regime. Such active metasurface can enable large TC difference between IMT. In the case of M1 phase, the TC of reflected light is −1 with average reflectivity of 70%; In the case of R phase, the TC of reflected light is −3 with averaged reflectivity of 68%. In addition, to show the flexibility of our method, two metasurfaces with TC of −2 (M1 phase) to −3 (R phase) and 4 (M1 phase) and −1 (R phase) are also presented. The proposed scheme enables more flexible design of the active metasurface, meanwhile empowering it with rich phase switching capability, instead of binary phase switching [58]. Our designed metasurfaces can be further utilized to study non‐Hermitian systems [60] or characterized by using photon emission electron microscope [61, 62]. It shows great promise in optical communication and dynamic display.

## 2 Materials and Methods

### 2.1 Theoretical Analysis

The reflection matrix of meta‐atoms after being rotated by an angle can be expressed as follows:

(1) $$R\left(\theta \right)=\left[\begin{array}{cc} \cos\;\theta & -\sin\;\theta \\ \sin\;\theta & \cos\;\theta \end{array}\right]\left[\begin{array}{cc} r_{xx} & r_{xy}\\ r_{yx} & r_{yy} \end{array}\right]\left[\begin{array}{cc} \cos\;\theta & \sin\;\theta \\ -\sin\;\theta & \cos\;\theta \end{array}\right],$$

where $r_{ij}$ is the reflection coefficient of *i*‐polarization under the *j*‐polarized normal incidence. By further applying R(θ) to normalized CP incidence, we can derive the Jones vector of the reflected light:

(2) $$J\left(\theta \right)=R\left(\theta \right)\left[\begin{array}{l} 1\\ \pm i \end{array}\right]=Ae^{i\phi_{\text{co}}}\left[\begin{array}{l} 1\\ \pm i \end{array}\right]+Be^{i\phi_{\text{cross}}}e^{\pm i2\theta }\left[\begin{array}{l} 1\\ \mp i \end{array}\right],$$

where $Ae^{i\phi_{\text{co}}}=\frac{1}{2}\left[\left(r_{xx}+r_{yy}\right)\pm \left(r_{xy}-r_{yx}\right)\right]$ and $Be^{i\phi_{\text{cross}}}=\frac{1}{2}\left[\left(r_{xx}-r_{yy}\right)\mp \left(r_{xy}-r_{yx}\right)\right]$ are co and cross‐polarized reflection coefficients determined by the structure itself. In addition, the reflected cross‐polarized beam carries the PB phase 2θ in Equation (2). Therefore, by further selecting element rotation angles, it is possible to generate vortex beams with decoupled TCs within a single metasurface.

In order to achieve different TCs in the same structure, we assume the phase distribution satisfies the expression below:

(3) $$\left\{\begin{array}{l} \phi_{1}+2\theta =l_{1}\varphi \\ \phi_{2}+2\theta =l_{2}\varphi \end{array}\right.,$$

which $l_{1}$ and $l_{2}$ refer to as the TCs possessed by the reflected beam, that is, the VO_2_ is in the insulating and metallic phase. φ represents the azimuthal angle at the transverse plane, and $\phi_{1}$ and $\phi_{2}$ stands for the propagation phase induced by the variations of the geometric parameters in the insulating and metallic phase, respectively. θ is the meta‐atom's rotation angle. Further considering the periodicity of phase modulation, the equations in Equation (3) evolve to Equation (4),

(4) $$\left\{\begin{array}{l} \phi_{1}+2\theta =\alpha_{1}\\ \phi_{2}+2\theta =\alpha_{2} \end{array}\right..$$

In the above equation, $\alpha_{i}=l_{i}\varphi +2k_{i}\pi$, in which $k_{i}\in Z$ and is a function of φ so as to restrain $\alpha_{i}$ to the range of. By solving Equation (4), the results are expressed in Equation (5).

(5) $$\left\{\begin{array}{l} \phi_{1}-\phi_{2}=\left(l_{1}-l_{2}\right)\varphi +2\pi \left(k_{1}-k_{2}\right)\\ \theta =\frac{l_{2}\phi_{1}-l_{1}\phi_{2}}{2\left(l_{1}-l_{2}\right)}+\pi \frac{l_{1}k_{2}-l_{2}k_{1}}{l_{1}-l_{2}} \end{array}\right..$$

**FIGURE 2:**
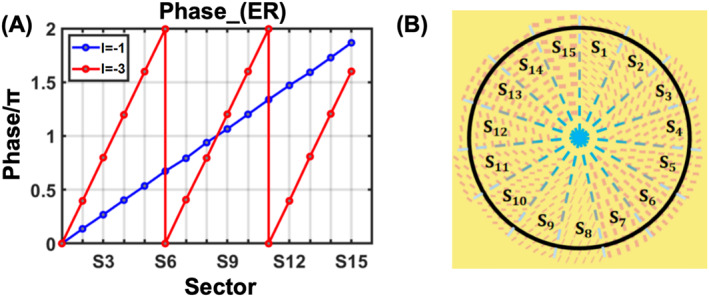
Phase modulation diagram and schematic diagram of metasurface arrangement. (A) Desired cross‐polarization phase shifts satisfying Equation (4) with respect to the azimuthal angle. (B) The schematic diagram of our proposed metasurface, which is divided into 15 sectors marked by *S*_*n*_.

Based on the above equation, it is possible to realize different helical phase distributions with the same structure. The first step is to get the difference of desired phase distribution at different azimuthal angles. Leveraging the phase difference distribution, we can assign the corresponding meta‐atom to specific azimuthal angle based on the phase difference database obtained through simulation. Each selected meta‐atom possesses distinct phase response in the M1 and R phase ($\phi_{1}$, $\phi_{2}$). Together with the designated TCs ($l_{1}$, $l_{2}$), we can further determine the required rotation angle θ of the corresponding meta‐atom to induce PB phase. In addition, $k_{1}$ and $k_{2}$ determined by restraining $\alpha_{i}$ to the range of [0, 2π] in Equation (4) should also be taken in to account. Thus, by considering periodicity of phase, higher TCs of OAM between IMT can be achieved. For demonstration, the ideal phase distribution when $l_{1}$ = −1 and $l_{2}$ = −3 is calculated based on $\alpha_{i}$ in Equation (4) and is presented in Figure 2A. Such desired phase response serves as a comparison against simulation results of meta‐atoms with selected phase difference and rotation angles, which is presented in Figure 2A. The schematic diagram of our proposed metasurface is shown in Figure 2B, the whole metasurface is divided into 15 sectors marked by *S*_*n*_.

### 2.2 The Design of Active Metasurface

The active metasurface simulations are carried out via the finite‐difference time‐domain (FDTD) method (Lumerical Inc.), whose TC of the reflected beam is −1 in the M1 phase and −3 in the R phase. The active metasurface whose TC changes from −2 to −3 is also simulated to demonstrate the flexibility of our proposed strategy. To engender large phase contrast, we adopted the aforementioned metal‐insulator‐metal (MIM) structure due to its deep subwavelength dimensions are capable of confining light inside VO_2_ with enhanced light–matter interaction in the M1 phase [63, 64]. It is a sandwich‐like structure with 100‐nm thick Au film as its top layer (to annihilate the direct coupling between the incident light and VO_2_ layer [63]), 100‐nm thick VO_2_ as the middle layer, and 2 μm‐thick flat Au layer underneath VO_2_ to enhance the reflection. Notably, in metal‐dielectric composite structures resembling our MIM structure, VO_2_'s plasmonic hot‐electron‐assisted IMT features an ultrafast switching time of 650 fs [47]. The refractive index of VO_2_ adopted in our simulations is presented in Supporting Information S1: Figure S4.

**FIGURE 3:**
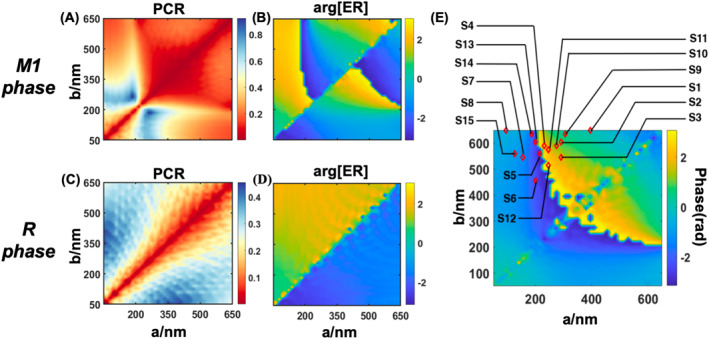
(A, C) Simulated cross‐polarization conversion rate with respect to the length and width of the meta‐atom in the M1 (A) and R (C) phase of VO_2_ for the converted RCP light with LCP incidence. (B, D) Simulated reflected phase response with respect to the length and width of the meta‐atom in the M1 (B) and R (D) phase of VO_2_ for the converted RCP light with LCP incidence. (E) The phase difference before and after IMT ($\Delta \phi =\phi_{1}-\phi_{2}$). Every red rhombus corresponds to the geometric parameters chosen for the corresponding sector.

**FIGURE 4:**
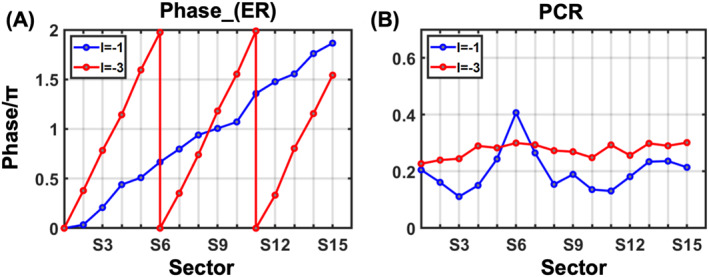
(A) Simulated cross‐polarization phase shifts satisfying Equation (4). (B) Simulated PCR for the selected meta‐atoms with respect to the azimuthal angle discretized into 15 sectors.

The lattice constant is set to 1.4 times the length of the structural diagonal to guarantee the meta‐atoms filling different sectors possess the same duty ratio despite different geometry. Subsequently, we sweep the width and length of the structure while the wavelength of the incident beam is set to 1500 nm. Considering the term of PB‐phase in Equation (1) is positive, the incident wave is set as left‐hand circularly polarized (LCP) light and the simulation results of PCR and phase response with respect to the parameters in the M1 and *R* phase of VO_2_ are depicted in Figure 3. (See Supporting Information S1: Note 5 for discussion on dual circular or unpolarized illumination). It is evident that the phase response of reflected light that corresponds to $\phi_{1}$ and $\phi_{2}$ in Equation (4) is distinctive before and after IMT. The difference can sufficiently cover the scope of 2π as shown in Figure 4. Such difference is regulated to the range of [−π,π).The open red rhombuses indicate each sector's selected parameters for the desired phase difference response.

For the ease of selection, we discretized $\varphi_{n}$ into 15 sections, each corresponding to a distinct meta‐atom with different dimensions marked by an open red rhombus in Figure 4, obtained judiciously from the simulation result. To select the required parameters, the phase difference of the chosen coefficients should satisfy Equation (5), whereas pursing uniform PCR response across sectors. (see Supporting Information S1: Note 1 for specific details)

We further rotated the selected pillar of different sectors by a specific angle to achieve desired vortex phase distribution. The resulting summation of the dynamic phase and PB phase is illustrated in Figure 4A. It shows one (three) abrupt phase changes while φ increasing from 0 to 2π in M1 (R) phase, indicating the simulation results agree well with our theoretical derivation in Figure 2A. The simulated PCR of the 15 selected meta‐atom is shown in Figure 4B. These results demonstrates that the selected parameters in Figure 3E are capable of generating TC of −1 and −3, with relatively consistent polarization conversion ratio (PCR) across sectors.

Using the selected meta‐atom, we now can construct the active metasurfaces. Since the azimuthal angle φ is divided into 15 sections, we fill the sectors by arranging the corresponding meta‐atom along the azimuthal and radial direction with the same duty ratio. This operation can introduce uniform phase modulation in the sectors and uniform phase increment. The consequent metasurface is illustrated in Figure 2D, and the simulation result of the metasurface is shown below.

## 3 Results and Discussion

**FIGURE 5:**
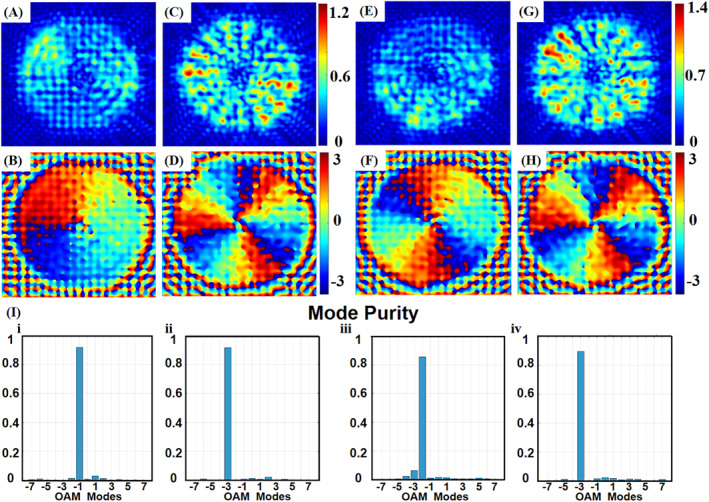
The far‐field simulation results of the proposed metasurface with −1 to −3 and −2 to −3 OAM transitions. (A–D) correspond to the intensity and phase distribution of the cross‐polarized light in the M1 phase, whereas (E–H) correspond to the intensity and phase distribution of the cross‐polarized light in the R phase. (I) Demonstrated the mode purity of the corresponding OAM modes. It is clearly shown that the mode purity is above 85% in all cases.

Under LCP incidence, the reflected phase profiles and intensity distributions are procured and plotted in Figure 5A–D. The spiral phase distribution of the reflected light implies the TC in the M1 (R) phase is −1 (−3), verifying that not only the active control of TCs is achieved but also the large TC difference is generated by incorporating VO_2_. In addition, to further demonstrate the flexibility of our scheme, we constructed the second and third active metasurface with distinct topological charge switching ability. The second metasurface switches between TC = −2 (M1 phase) and TC = −3 (R phase; see Supporting Information S1: Note 2), whereas the third metasurface switches between TC = 4 (M1 phase) and TC = −1 (R phase; see Supporting Information S1: Note 6). The simulation results of the second metasurface are shown in Figure 5E–H, whereas the OAM spectra of each generated OAM mode for the first and the second metasurface is shown in Figure 5I. The first design achieves mode purities of 91.9% (M1) and 91.2% (R), with a reflectivity of ∼70% and PCR of ∼20% (M1) and ∼30% (R). The second yields 85.6% (M1) and 89.6% (R) mode purity, with ∼70% reflectivity and similar PCR values. The output intensity uniformity can be further enhanced by optimizing the meta‐atom duty ratio. In addition, the reflectivity can also be enhanced by further reducing the thickness of Au topping or VO_2_ layers to reduce the absorption. For further experimental realizations, the MIM structures with 100 nm thick VO_2_ can be easily fabricated by using electron beam lithography (EBL), magnetron sputtering, or molecular beam epitaxy. At last, utilizing VO_2_ in the metasurfaces provides numerous ways to modulate actively. Beyond heating and electric field excitation, femtosecond laser pulses can induce VO_2_'s phase transition featuring a sub‐picosecond switching time, which paves the way for probing the femtosecond‐scale dynamics of OAM transitions using pump‐probe techniques [65] (See Supporting Information S1: Note 4 for an additional discussion on the thermal crosstalk of VO_2_ integrated metasurface).

## 4 Conclusion

In summary, we have proposed and numerically demonstrated reflective active metasurfaces that leverages the IMT of VO_2_ to dynamically switch between distinct orbital angular momentum (OAM) emission. By strategically integrating VO_2_ as the functional core of MIM meta‐atoms and judiciously combining the propagation and geometric phases based on our proposed strategy, we achieved a significant switching of the topological charge (e.g., from ℓ = −1 to ℓ = −3) in the telecommunications band. This design fully capitalizes on the substantial refractive index contrast of VO_2_ during phase transition, moving beyond its conventional use as a thin layer. Our approach offers a versatile and fabrication‐friendly platform for active photonics in the near‐infrared, promising applications in high‐capacity OAM multiplexing. Furthermore, the compatibility of VO_2_ with ultrafast optical excitation opens a pathway for future investigations into ultrafast OAM modulation dynamics.

## Author Contributions

Qinghong Lyu performed the numerical simulations. Qiuchen Yan helped with the theoretical analysis. Yulan Fu, Xiaoyong Hu, and Qihuang Gong supervised the work. Qinghong Lyu wrote the original draft. All authors were involved in the discussion and analysis. All authors have accepted responsibility for the entire content of this manuscript and consented to its submission to the journal and reviewed all the results.

## Funding

This work was supported by the National Key Research and Development Program of China under Grant No. 2024YFA1209204, National Natural Science Foundation of China under Grant Nos. 12474322, 12404422, and 62405211, Quantum Science and Technology‐National Science and Technology Major Project under Grant No. 2021ZD0301500, Fundamental and Interdisciplinary Disciplines Breakthrough Plan of the Ministry of Education of China under Grant No. JYB2025XDXM106, Beijing Natural Science Foundation under Grant No. 1262029, China Postdoctoral Science Foundation (2023M740121 and 2024T170008), and Beijing Municipal Education Commission (KM202410005015).

## Conflicts of Interest

The authors declare no conflicts of interest.

## Supporting information


Supporting Information S1

## Data Availability

All data are available in the main text, supplementary materials.

## References

[1] L. Allen , S. M. Barnett , and M. J. Padgett , Optical Angular Momentum (CRC Press, 2016).

[2] J. P. Torres and L. Torner , Twisted Photons: Applications of Light With Orbital Angular Momentum (John Wiley & Sons, 2011).

[3] L. Allen , M. W. Beijersbergen , R. Spreeuw , and J. Woerdman , “Orbital Angular Momentum of Light and the Transformation of Laguerre‐Gaussian Laser Modes,” Physical Review A 45, no. 11 (1992): 8185–8189, 10.1103/physreva.45.8185. 9906912

[4] J. Chen , C. Wan , and Q. Zhan , “Engineering Photonic Angular Momentum With Structured Light: A Review,” Advanced Photonics 3, no. 6 (2021): 064001, 10.1117/1.ap.3.6.064001.

[5] J. Wang , J.‐Y. Yang , I. M. Fazal , et al., “Terabit Free‐Space Data Transmission Employing Orbital Angular Momentum Multiplexing,” Nature Photonics 6, no. 7 (2012): 488–496, 10.1038/nphoton.2012.138.

[6] N. Bozinovic , Y. Yue , Y. Ren , et al., “Terabit‐Scale Orbital Angular Momentum Mode Division Multiplexing in Fibers,” Science 340, no. 6140 (2013): 1545–1548, 10.1126/science.1237861. 23812709

[7] S. W. Hell and J. Wichmann , “Breaking the Diffraction Resolution Limit by Stimulated Emission: Stimulated‐Emission‐Depletion Fluorescence Microscopy,” Optics Letters 19, no. 11 (1994): 780–782, 10.1364/ol.19.000780. 19844443

[8] M. E. Friese , T. A. Nieminen , N. R. Heckenberg , and H. Rubinsztein‐Dunlop , “Optical Alignment and Spinning of Laser‐Trapped Microscopic Particles,” Nature 394, no. 6691 (1998): 348–350, 10.1038/28566.

[9] V. Y. Bazhenov , M. Vasnetsov , and M. Soskin , “Laser Beams With Screw Dislocations in Their Wavefronts,” JETP Letters 52, no. 8 (1990): 429–431, 10.1887/0750309016/b1142c24.

[10] M. Beijersbergen , R. Coerwinkel , M. Kristensen , and J. Woerdman , “Helical‐Wavefront Laser Beams Produced With a Spiral Phaseplate,” Optics Communications 112, no. 5–6 (1994): 321–327, 10.1016/0030-4018(94)90638-6.

[11] L. Marrucci , C. Manzo , and D. Paparo , “Optical Spin‐to‐Orbital Angular Momentum Conversion in Inhomogeneous Anisotropic Media,” Physical Review Letters 96, no. 16 (2006): 163905, 10.1103/physrevlett.96.163905. 16712234

[12] M. W. Beijersbergen , L. Allen , H. Van der Veen , and J. Woerdman , “Astigmatic Laser Mode Converters and Transfer of Orbital Angular Momentum,” Optics Communications 96, no. 1–3 (1993): 123–132, 10.1016/0030-4018(93)90535-d.

[13] A. V. Kildishev , A. Boltasseva , and V. M. Shalaev , “Planar Photonics With Metasurfaces,” Science 339, no. 6125 (2013): 1232009, 10.1126/science.1232009. 23493714

[14] N. Yu , P. Genevet , M. A. Kats , et al., “Light Propagation With Phase Discontinuities: Generalized Laws of Reflection and Refraction,” Science 334, no. 6054 (2011): 333–337, 10.1126/science.1210713. 21885733

[15] A. Arbabi , Y. Horie , M. Bagheri , and A. Faraon , “Dielectric Metasurfaces for Complete Control of Phase and Polarization With Subwavelength Spatial Resolution and High Transmission,” Nature Nanotechnology 10, no. 11 (2015): 937–943, 10.1038/nnano.2015.186. 26322944

[16] C. Chen , S. Gao , W. Song , H. Li , S.‐N. Zhu , and T. Li , “Metasurfaces With Planar Chiral Meta‐Atoms for Spin Light Manipulation,” Nano Letters 21, no. 4 (2021): 1815–1821, 10.1021/acs.nanolett.0c04902. 33533621

[17] K. T. Lim , H. Liu , Y. Liu , and J. K. Yang , “Holographic Colour Prints for Enhanced Optical Security by Combined Phase and Amplitude Control,” Nature Communications 10, no. 1 (2019): 1–8, 10.1038/s41467-018-07808-4. PMC6318302 30604762

[18] R. C. Devlin , A. Ambrosio , N. A. Rubin , J. B. Mueller , and F. Capasso , “Arbitrary Spin‐to‐Orbital Angular Momentum Conversion of Light,” Science 358, no. 6365 (2017): 896–901, 10.1126/science.aao5392. 29097490

[19] Y. Guo , M. Pu , Z. Zhao , et al., “Merging Geometric Phase and Plasmon Retardation Phase in Continuously Shaped Metasurfaces for Arbitrary Orbital Angular Momentum Generation,” ACS Photonics 3, no. 11 (2016): 2022–2029, 10.1021/acsphotonics.6b00564.

[20] K. Yang , M. Pu , X. Li , et al., “Wavelength‐Selective Orbital Angular Momentum Generation Based on a Plasmonic Metasurface,” Nanoscale 8, no. 24 (2016): 12267–12271, 10.1039/c5nr09209d. 27271957

[21] M. Pu , X. Li , X. Ma , et al., “Catenary Optics for Achromatic Generation of Perfect Optical Angular Momentum,” Science Advances 1, no. 9 (2015): e1500396, 10.1126/sciadv.1500396. 26601283 PMC4646797

[22] Y. Guo , S. Zhang , M. Pu , et al., “Spin‐Decoupled Metasurface for Simultaneous Detection of Spin and Orbital Angular Momenta via Momentum Transformation,” Light: Science & Applications 10, no. 1 (2021): 63, 10.1038/s41377-021-00497-7. PMC7994415 33767137

[23] Z. Zhang , G. Li , Y. Liu , et al., “Robust Measurement of Orbital Angular Momentum of a Partially Coherent Vortex Beam Under Amplitude and Phase Perturbations,” Opto‐Electronic Science 3, no. 1 (2024): 240001–240011, 10.29026/oes.2024.240001.

[24] H. Yang , S. Zheng , H. Zhang , et al., “A THz‐OAM Wireless Communication System Based on Transmissive Metasurface,” IEEE Transactions on Antennas and Propagation 71, no. 5 (2023): 4194–4203, 10.1109/tap.2023.3255539.

[25] A. Ali , M. Khalily , D. Serghiou , and R. Tafazolli , “Reflective Metasurface With Steered OAM Beams for THz Communications,” IEEE Access 11 (2023): 12394–12401, 10.1109/access.2023.3242647.

[26] L. Kang , R. P. Jenkins , and D. H. Werner , “Recent Progress in Active Optical Metasurfaces,” Advanced Optical Materials 7, no. 14 (2019): 1801813, 10.1002/adom.201801813.

[27] T. Cui , B. Bai , and H. B. Sun , “Tunable Metasurfaces Based on Active Materials,” Advanced Functional Materials 29, no. 10 (2019): 1806692, 10.1002/adfm.201806692.

[28] A. M. Shaltout , V. M. Shalaev , and M. L. Brongersma , “Spatiotemporal Light Control With Active Metasurfaces,” Science 364, no. 6441 (2019): eaat3100, 10.1126/science.aat3100. 31097638

[29] B. Kang , J. H. Woo , E. Choi , et al., “Optical Switching of Near Infrared Light Transmission in Metamaterial‐Liquid Crystal Cell Structure,” Optics Express 18, no. 16 (2010): 16492–16498, 10.1364/oe.18.016492. 20721037

[30] A. Komar , R. Paniagua‐Domínguez , A. Miroshnichenko , et al., “Dynamic Beam Switching by Liquid Crystal Tunable Dielectric Metasurfaces,” ACS Photonics 5, no. 5 (2018): 1742–1748, 10.1021/acsphotonics.7b01343.

[31] S.‐Q. Li , X. Xu , R. Maruthiyodan Veetil , V. Valuckas , R. Paniagua‐Domínguez , and A. I. Kuznetsov , “Phase‐Only Transmissive Spatial Light Modulator Based on Tunable Dielectric Metasurface,” Science 364, no. 6445 (2019): 1087–1090, 10.1126/science.aaw6747. 31197013

[32] Z. Miao , Q. Wu , X. Li , et al., “Widely Tunable Terahertz Phase Modulation With Gate‐Controlled Graphene Metasurfaces,” Physical Review X 5, no. 4 (2015): 041027, 10.1103/physrevx.5.041027.

[33] Z. Su , F. Cheng , L. Li , and Y. Liu , “Complete Control of Smith‐Purcell Radiation by Graphene Metasurfaces,” ACS Photonics 6, no. 8 (2019): 1947–1954, 10.1021/acsphotonics.9b00251.

[34] W. Ma , Z. Huang , X. Bai , P. Zhan , and Y. Liu , “Dual‐Band Light Focusing Using Stacked Graphene Metasurfaces,” ACS Photonics 4, no. 7 (2017): 1770–1775, 10.1021/acsphotonics.7b00351.

[35] Z.‐Y. Jia , F.‐Z. Shu , Ya‐J. Gao , et al., “Dynamically Switching the Polarization State of Light Based on the Phase Transition of Vanadium Dioxide,” Physical Review Applied 9, no. 3 (2018): 034009, 10.1103/physrevapplied.9.034009.

[36] Y. Meng , J. K. Behera , Y. Ke , et al., “Design of a 4‐Level Active Photonics Phase Change Switch Using VO_2_ and Ge_2_Sb_2_Te_5_ ,” Applied Physics Letters 113, no. 7 (2018): 071901, 10.1063/1.5043521.

[37] K. V. Sreekanth , S. Han , and R. Singh , “Ge_2_Sb_2_Te_5_‐Based Tunable Perfect Absorber Cavity With Phase Singularity at Visible Frequencies,” Advanced Materials 30, no. 21 (2018): 1706696, 10.1002/adma.201706696. 29635805

[38] F. Zhang , X. Xie , M. Pu , et al., “Multistate Switching of Photonic Angular Momentum Coupling in Phase‐Change Metadevices,” Advanced Materials 32, no. 39 (2020): 1908194, 10.1002/adma.201908194. 32851702

[39] Z. Cai , C. Wu , J. Jiang , Y. Ding , Z. Zheng , and F. Ding , “Phase‐Change Metasurface for Switchable Vector Vortex Beam Generation,” Optics Express 29, no. 26 (2021): 42762–42771, 10.1364/oe.444956.

[40] F. Morin , “Oxides Which Show a Metal‐to‐Insulator Transition at the Neel Temperature,” Physical Review Letters 3, no. 1 (1959): 34–36, 10.1103/physrevlett.3.34.

[41] J. Del Valle , P. Salev , F. Tesler , et al., “Subthreshold Firing in Mott Nanodevices,” Nature 569, no. 7756 (2019): 388–392, 10.1038/s41586-019-1159-6. 31043748

[42] F. Z. Shu , J.‐N. Wang , R.‐W. Peng , et al., “Electrically Driven Tunable Broadband Polarization States via Active Metasurfaces Based on Joule‐Heat‐Induced Phase Transition of Vanadium Dioxide,” Laser & Photonics Reviews 15, no. 10 (2021): 2100155, 10.1002/lpor.202100155.

[43] J. Cao , E. Ertekin , V. Srinivasan , et al., “Strain Engineering and One‐Dimensional Organization of Metal–Insulator Domains in Single‐Crystal Vanadium Dioxide Beams,” Nature Nanotechnology 4, no. 11 (2009): 732–737, 10.1038/nnano.2009.266. 19893528

[44] D. Wegkamp , M. Herzog , L. Xian , et al., “Instantaneous Band Gap Collapse in Photoexcited Monoclinic VO_2_ due to Photocarrier Doping,” Physical Review Letters 113, no. 21 (2014): 216401, 10.1103/physrevlett.113.216401. 25479507

[45] V. R. Morrison , R. P. Chatelain , K. L. Tiwari , et al., “A Photoinduced Metal‐Like Phase of Monoclinic VO_2_ Revealed by Ultrafast Electron Diffraction,” Science 346, no. 6208 (2014): 445–448, 10.1126/science.1253779. 25342797

[46] M. F. Jager , C. Ott , P. M. Kraus , et al., “Tracking the Insulator‐to‐Metal Phase Transition in VO_2_ With Few‐Femtosecond Extreme UV Transient Absorption Spectroscopy,” Proceedings of the National Academy of Sciences 114, no. 36 (2017): 9558–9563, 10.1073/pnas.1707602114. PMC5594684 28827356

[47] Y. Fu , Z. Song , M. Jiang , et al., “Plasmonic Hot‐Electron Injection Driving Ultrafast Phase Transition in Self‐Supported VO_2_ Films for All‐Optical Modulation,” ACS Photonics 9, no. 12 (2022): 3950–3957, 10.1021/acsphotonics.2c01326.

[48] L. Liu , L. Kang , T. S. Mayer , and D. H. Werner , “Hybrid Metamaterials for Electrically Triggered Multifunctional Control,” Nature Communications 7, no. 1 (2016): 1–8, 10.1038/ncomms13236. PMC5095288 27807342

[49] Y. Kim , P. C. Wu , R. Sokhoyan , et al., “Phase Modulation With Electrically Tunable Vanadium Dioxide Phase‐Change Metasurfaces,” Nano Letters 19, no. 6 (2019): 3961–3968, 10.1021/acs.nanolett.9b01246. 31136191

[50] S. K. Earl , T. D. James , D. E. Gomez , R. E. Marvel , R. F. Haglund Jr. , and A. Roberts , “Switchable Polarization Rotation of Visible Light Using a Plasmonic Metasurface,” APL Photonics 2, no. 1 (2017): 016103, 10.1063/1.4968840.

[51] X. Li , S. Tang , F. Ding , et al., “Switchable Multifunctional Terahertz Metasurfaces Employing Vanadium Dioxide,” Scientific Reports 9, no. 1 (2019): 1–13, 10.1038/s41598-019-41915-6. 30931982 PMC6443649

[52] W. Chen , R. Chen , Y. Zhou , R. Chen , and Y. Ma , “Spin‐Dependent Switchable Metasurfaces Using Phase Change Materials,” Optics Express 27, no. 18 (2019): 25678–25687, 10.1364/oe.27.025678. 31510436

[53] R. Nie , C. He , R. Zhang , and Z. Song , “Vanadium Dioxide‐Based Terahertz Metasurfaces for Manipulating Wavefronts With Switchable Polarization,” Optics & Laser Technology 159 (2023): 109010, 10.1016/j.optlastec.2022.109010.

[54] K. Guo , J. Xin , and Z. Song , “Terahertz Six‐Channel Metasurface for the Dynamic Modulation of OAM,” Journal of Physics D Applied Physics 58, no. 6 (2025): 065109, 10.1088/1361-6463/ad9485.

[55] J. Zhan , Y. Zhang , Q. Li , and Z. Song , “Broadband Multifunctional Metasurface for Dynamic Wavefront Modulation,” Optics and Lasers in Engineering 194 (2025): 109193, 10.1016/j.optlaseng.2025.109193.

[56] M. Liu , J. Yang , Z. Du , J. Xin , and Z. Song , “Tripolarization‐Channel Holograms Generated by Terahertz Reflective Bilayer‐Metasurface,” Optics and Lasers in Engineering 186 (2025): 108763, 10.1016/j.optlaseng.2024.108763.

[57] J. Yang , Z. Xu , J. Xin , Z. Song , “Temperature‐Assisted Terahertz Reconfigurable Metasurface for Multi‐Polarization Holographic Display and Encryption” Optics & Laser Technology 181 (2025): 111968, 10.1016/j.optlastec.2024.111968.

[58] Y. Liao , Y. Fan , and D. Lei , “Thermally Tunable Binary‐Phase VO_2_ Metasurfaces for Switchable Holography and Digital Encryption,” Nanophotonics 13, no. 7 (2024): 1109–1117, 10.1515/nanoph-2023-0824. 39634017 PMC11501404

[59] B. Dong , S. Zhu , G. Guo , et al., “Switchable Pancharatnam‐Berry Phases in Heterogeneously Integrated THz Metasurfaces,” Advanced Materials 37, no. 6 (2025): 2417183, 10.1002/adma.202417183. 39676492

[60] Q. Yan , B. Zhao , R. Zhou , et al., “Advances and Applications on Non‐Hermitian Topological Photonics,” Nanophotonics 12, no. 13 (2023): 2247–2271, 10.1515/nanoph-2022-0775. 39633755 PMC11501638

[61] Q. Yan , En Cao , Q. Sun , et al., “Near‐Field Imaging and Time‐Domain Dynamics of Photonic Topological Edge States in Plasmonic Nanochains,” Nano Letters 21, no. 21 (2021): 9270–9278, 10.1021/acs.nanolett.1c03324. 34670093

[62] Q. Yan , B. Zhao , Q. Lyu , et al., “Near‐Field Imaging of Synthetic Dimensional Integrated Plasmonic Topological Harper Nanochains,” Nature Communications 16, no. 1 (2025): 2592, 10.1038/s41467-025-57747-0. PMC11911440 40091089

[63] J. Zhang , Y. Kosugi , A. Otomo , et al., “Electrical Tuning of Metal‐Insulator‐Metal Metasurface With Electro‐Optic Polymer,” Applied Physics Letters 113, no. 23 (2018): 231102, 10.1063/1.5054964.

[64] J. Kim , E. G. Carnemolla , C. DeVault , et al., “Dynamic Control of Nanocavities With Tunable Metal Oxides,” Nano Letters 18, no. 2 (2018): 740–746, 10.1021/acs.nanolett.7b03919. 29283583

[65] J. Du , Z. Mu , L. Li , and J. Li , “A Raman Study on Nanosecond‐Laser‐Induced Multi‐Level Switching of Ge_2_Sb_2_Te_5_ Thin Films,” Optics & Laser Technology 144 (2021): 107393, 10.1016/j.optlastec.2021.107393.

