# Supplementary material for: Active Switching of Orbital Angular Momentum of Light Using Metasurfaces Incorporating Vanadium Dioxide
Source: Nanophotonics. 2026 Feb 26;15(5):e70040. doi: 10.1002/nap2.70040 (PMC12965009; doi:10.1002/nap2.70040)
Supplement: Supplementary file 1 — Supporting Information S1 [file NAP2-15-e70040-s001.docx]

Supporting Materials

Active switching of orbital angular momentum of light using metasurfaces incorporating vanadium dioxide

*Qinghong Lyu^1,#^, Qiuchen Yan^1,#^,*, Yulan Fu^2^,*, Xiaoyong Hu^1,3,4,5^,*, Qihuang Gong^1,3,4,5^*

**Affiliations:**

^1^State Key Laboratory for Mesoscopic Physics & Department of Physics, Collaborative Innovation Center of Quantum Matter & Frontiers Science Center for Nano-optoelectronics, Peking University, Beijing 100871, China

^2^Institute of Information Photonics Technology, School of Physics and Optoelectronic Engineering, Beijing University of Technology, Beijing100124, P.R. China

^3^Key Laboratory for Advanced Optoelectronic Integrated Chips of Jiangsu Province， Peking University Yangtze Delta Institute of Optoelectronics, Nantong, Jiangsu 226010, China

^4^Collaborative Innovation Center of Extreme Optics, Shanxi University, Taiyuan, Shanxi 030006, China.

^5^Hefei National Laboratory, Hefei 230088, China

^#^ The authors contributed equally to this work.

* Corresponding authors: [qiuchenyan@pku.edu.cn](mailto:qiuchenyan@pku.edu.cn); fuyl@bjut.edu.cn; xiaoyonghu@pku.edu.cn.

**Contents**

Supplementary Note 1: Geometric parameter selection

Supplementary Note 2: Designing the second metasurface

Supplementary Note 3: The Refractive index of VO_2_

Supplementary Note 4: The discussion of thermal crosstalk

Supplementary Note 5: Active OAM switching for dual circular and unpolarized incidence

Supplementary Note 6: The design and performance of -1-4 OAM mode switching

**Supplementary Note 1: Geometric parameter selection**

Two-dimensional parameter sweeps were done with the width and length of the structure varying from 50 nm to 650 nm with a step of 15 nm. Considering the fact that different meta-atom filling the corresponding sector possesses distinct duty ratio, the lattice constant (d) was set to vary together with the geometric dimension of the meta-atom. We apply the following equation to set the lattice constant for each meta-atom:

$d=c\cdot\sqrt{a^{2}+b^{2}}$ (1)

where a represents width and b represents length, d is the lattice constant for the corresponding meta-atom. Parameter c is a constant that should not be too small, or else the units will overlap; on the other hand, the value of c should match the actual arrangement density. In our work, we set c to be 1.4. With the simulation results obtained, the phase response in the insulating and metallic state of VO_2_ in the parameter space, as well as the phase difference $\Delta\varphi=\varphi_{1}-\varphi_{2}$ is procured, as shown in Fig.3 in the main text. The metasurface is further divided into 15 sectors, as shown in Fig. 2 in the main text. For the k_th_ sector, which is labeled as S_k_, we set its azimuthal angle to be:

$\phi_{k}=\frac{k-1}{15}\cdot2\pi$ (2)


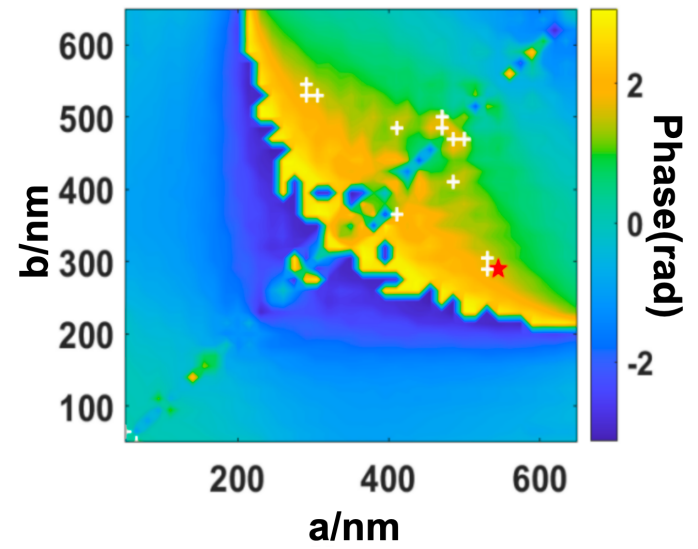


**Fig. S1.** The process that pin down the final meta-atom geometry within one sector. The candidate parameters that satisfy the phase difference requirement are marked down with labels. Only the red star is finally picked up judiciously for uniform PCR performances across sectors under both VO_2_ phases.

The optimal meta-atom for each sector was selected by screening all candidate structures for sector Sn whose phase difference fell within a 0.05 rad tolerance of the value calculated from Eq. (5). Among these candidates, the structure exhibiting better PCR uniformity across sectors under both VO₂ phases (M1 and R) was chosen. As illustrated in Fig. S1, where each pixel in the parameter space represents a unique combination (width a and length b). The candidate units for a representative sector are marked by white crosses. The final selection, indicated by a red star. The parameters for all selected meta-atoms across the metasurface are summarized in Table S1.

**Table S1.** The parameters of the meta-atoms for -1- -3 metasurface.

With the 15 units we have picked up, the rotation angle can thus be procured by taking the corresponding$\varphi_{1}$and$\varphi_{2}$of the selected units into Eq. (5) shown in the main text. Therefore, the final simulated phase response in the reflected cross-polarization can be obtained in Fig, S2(B), and the results agree well with the theoretical phase response (Fig. S2(A)) calculated based on Eq. (4). Based on such verification, we uniformly arranged these units in their corresponding sector with the same duty ratio, and the distances between these units were slightly tuned to improve the total performance.


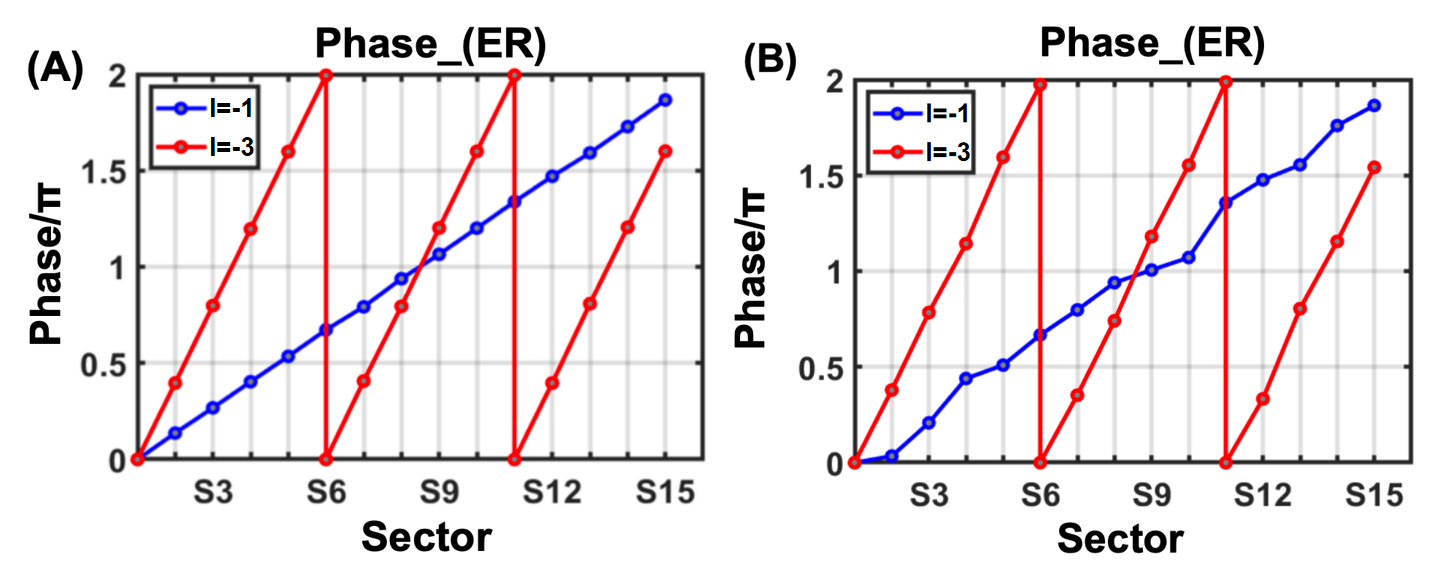


**Fig. S2.** Summation of dynamic phase and PB phase (a) and simulation phase (b) for 15 units that participate in constructing -1 - -3 metasurface.

**Supplementary Note 2: Designing the second metasurface**

To further demonstrate the flexibility of the proposed method, we applied the same procedure to construct another active metasurface whose topological charge could switch between -2 and -3. To begin with, 15 meta-atoms that correspond to 15 sectors of the metasurface were selected, which are marked by red hollowed rhombuses in Fig. S3(A), and their geometric parameters are shown in Table S2.

**Table S2.** The parameters of the meta-atom for -2- -3 metasurface.

In addition, we calculated the rotation angle$\theta$ according to Eq. (4) and drew the theoretical summation of dynamic phase and PB phase response in Fig. S3(C). Similar to Fig. S2(B), Fig. S3(D) shows the simulated total phase response when we rotate the selected units by the desired angle, it shows a significant similarity with theoretical summation result in Fig. S3(C). Finally, the metasurface was arranged in the same way as we introduced in the main text, and it successfully generated the vortex beams we desired in different phase of VO2, as shown in Fig. 5(E-F) in the main text, despite moderate discrepancy of PCR across sectors shown in Fig.S3(E). The figure of such metasurface is demonstrated in Fig. S3(B).


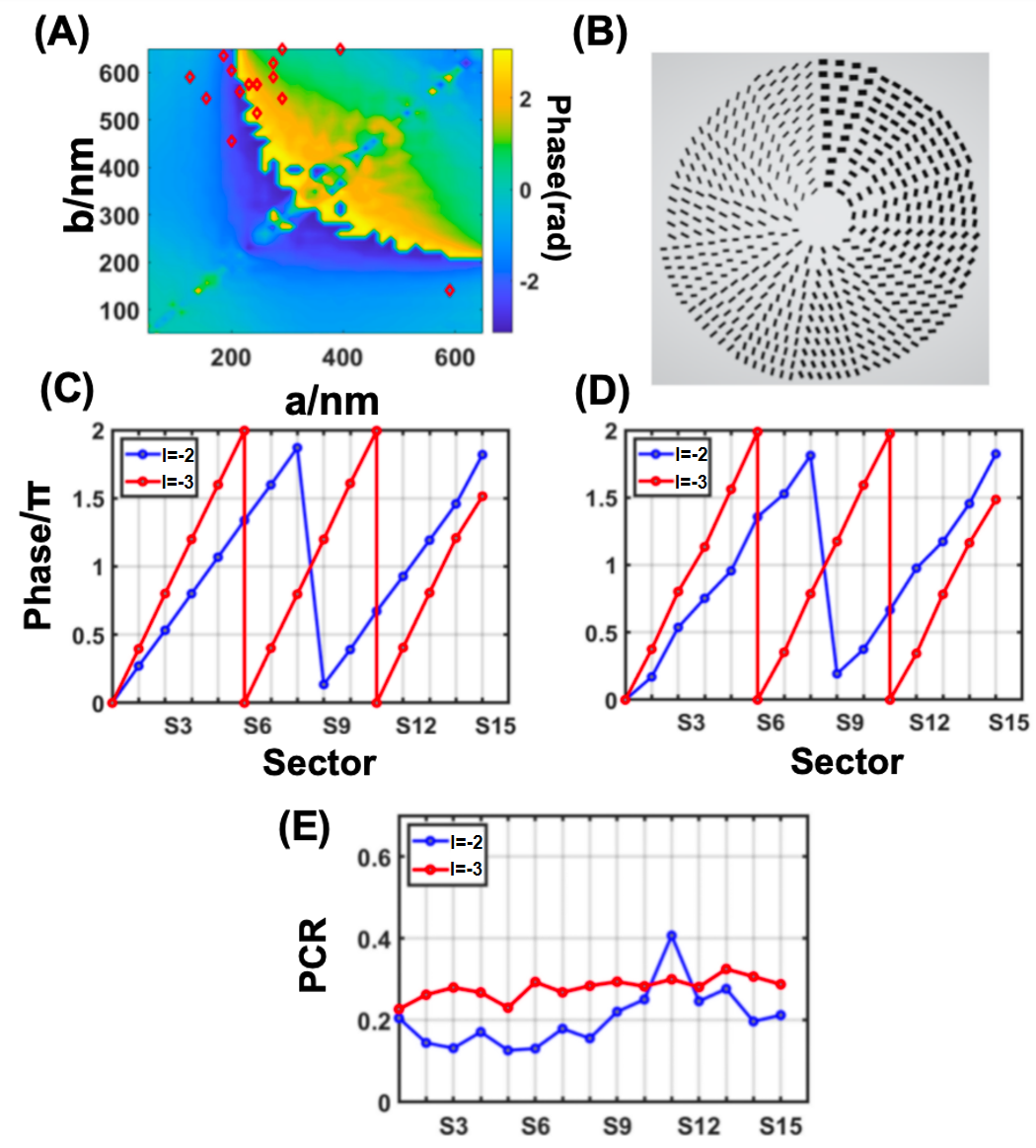


**Fig. S3.** **Construction Information for the -2- -3 metasurface.** (a) Selected 15 units to construct the metasurface, marked by red hollowed rhombuses. (b) Diagram of -2- -3 metasurface. Summation of dynamic phase and PB phase (c) and simulation phase (d) for 15 units that participate in constructing -2- -3 metasurface. (e) PCR with respect to the corresponding sector.

**Supplementary Note 3: The Refractive index of VO_2_**

**
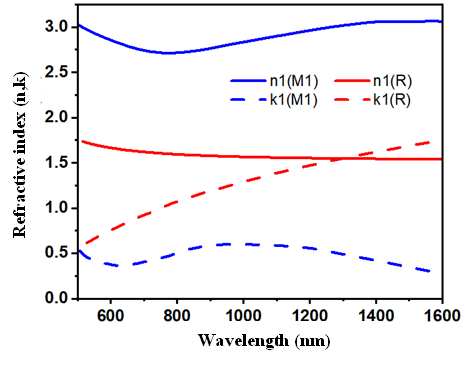
**

**Fig. S4**. The refractive index of VO_2_ which we utilized for simulation. The blue lines indicate the refractive index in M1 phase, while the red lines indicate the refractive index in R phase.

**Supplementary Note 4: The discussion of thermal crosstalk**

Heating VO_2_ to experience phase transition is a practical way for real-world applications, however, local heating in an array may cause thermal crosstalk, thus managing heat diffusion is essential for achieving reliable and stable active switching in an array. To address this challenge, we now provide a detailed discussion on the suppression of thermal crosstalk in terms of thermal design, ultrafast excitation, and the intrinsic material properties of VO₂.

1. **Thermal Management through Structural Design：**
   The most direct approach is to engineer the thermal pathway of the device. This can be achieved by optimizing the vertical thermal interface between the VO₂ array and the heater. For instance, studies have shown that thermal conduction between the MIM structures and the heating stage is critical to suppress the thermal crosstalk. To achieve good thermal contact between the two objects, a layer composed of liquid metal, i.e. gallium-based alloy, was used to fill their gap, and the thermal crosstalk is suppressed [1].
2. **Ultrafast Optical Excitation to mitigate thermal crosstalk:**

A highly effective strategy to circumvent thermal diffusion is to use ultrafast (femtosecond or picosecond) laser pulses for triggering the phase transition. The energy deposition from such pulses occurs on a picosecond timescale, which is orders of magnitude shorter than the microsecond timescale required for heat dissipation[2]. This ensures that the phase transition is accomplished before significant thermal crosstalk can develop. This approach directly leverages the ultrafast photo-induced phase transition capability intrinsic to VO₂.

1. **Intrinsic Thermal Robustness of the VO₂ Metallic Phase:**Finally, our device benefits from a key material property: the refractive index and extinction coefficient of VO₂ remain stable above the phase transition temperature (>70°C)[3]. Once a local unit is switched into the metallic state, a minor temperature fluctuation in a neighboring unit (caused by thermal crosstalk) is unlikely to induce a significant change in its optical properties unless it crosses the phase transition threshold. Therefore, the device's functionality is defined by the insulating or metallic phase rather than by minute temperature gradients, providing inherent robustness against limited thermal crosstalk.

In summary, thermal crosstalk can be mitigated through a combination of strategic thermal engineering of the device layout, employing ultrafast optical triggering to outpace heat diffusion, and relying on the stable optical properties of VO₂'s metallic phase. These complementary approaches ensure that our proposed active metasurface platform can achieve reliable and crosstalk-suppressed dynamic switching.

**Supplementary Note 5: Active OAM switching for dual circular and unpolarized incidence**

While the main text focuses on the metasurface design under LCP incidence, we have extended the theoretical framework to enable OAM switching for both LCP and RCP illumination. In the following, we provide a detailed analysis of how our design principle could be extended to handle RCP and unpolarized light, along with the potential pathways to extend the OAM generation capabilities closer to real-world applications.

**1. Theoretical Extension to Dual-circular Polarization**

Our current design is optimized for LCP incidence by satisfying the following two equations that couple the propagation phase ($\varphi_{1}$, $\varphi_{2}$) and the PB phase ($2\theta$):

$\left\{ \begin{aligned} \varphi_{1}+2\theta=l_{1}\phi\\ \varphi_{2}+2\theta=l_{2}\phi\end{aligned} \right.$ (3)

where $\phi$ is the azimuthal coordinate on the metasurface, $l_{1}$ and $l_{2}$ are the desired topological charges for the insulating (M1) and metallic (R) phases of VO₂, respectively, under LCP illumination.

To achieve active OAM switching also for RCP incidence, the same metasurface would need to simultaneously satisfy two additional equations:

$\left\{ \begin{aligned} \varphi_{1}-2\theta=l_{3}\phi\\ \varphi_{2}-2\theta=l_{4}\phi\end{aligned} \right.$ (4)

where $l_{3}$  and $l_{4}$  are the target topological charges for the M1 and R phase of VO₂ under RCP illumination. The sign reversal of the geometric phase term (−$2\theta$) reflects the opposite spin-orbit interaction for RCP.

Combining the four equations (3) and (4) leads to a set of necessary conditions:

$\left\{ \begin{aligned} l_{1}-l_{2}=l_{3}-l_{4} \\ \varphi_{1}+\varphi_{2}=\frac{l_{1}+l_{2}+l_{3}+l_{4}}{2}\phi\\ \varphi_{1}-\varphi_{2}=(l_{1}-l_{2})\phi\\ \theta=\frac{l_{1}+l_{2}-l_{3}-l_{4}}{8}\phi\end{aligned} \right.$ (5)

The above Eq. (5) indicate that for each azimuthal angle$\phi$, we can select a meta-atom from the library such that the sum and difference of the propagation phases $\varphi_{1}$and$\varphi_{2}$satisfy the regulation that caused by the four distinct topological charges, and then assign the rotation angle$\theta$according to Eq. (5). Provided that the four topological charges satisfy condition described in Eq. (5), we can thus generate four different topological charges for LCP and RCP incidence in the insulating and metallic phases, respectively.


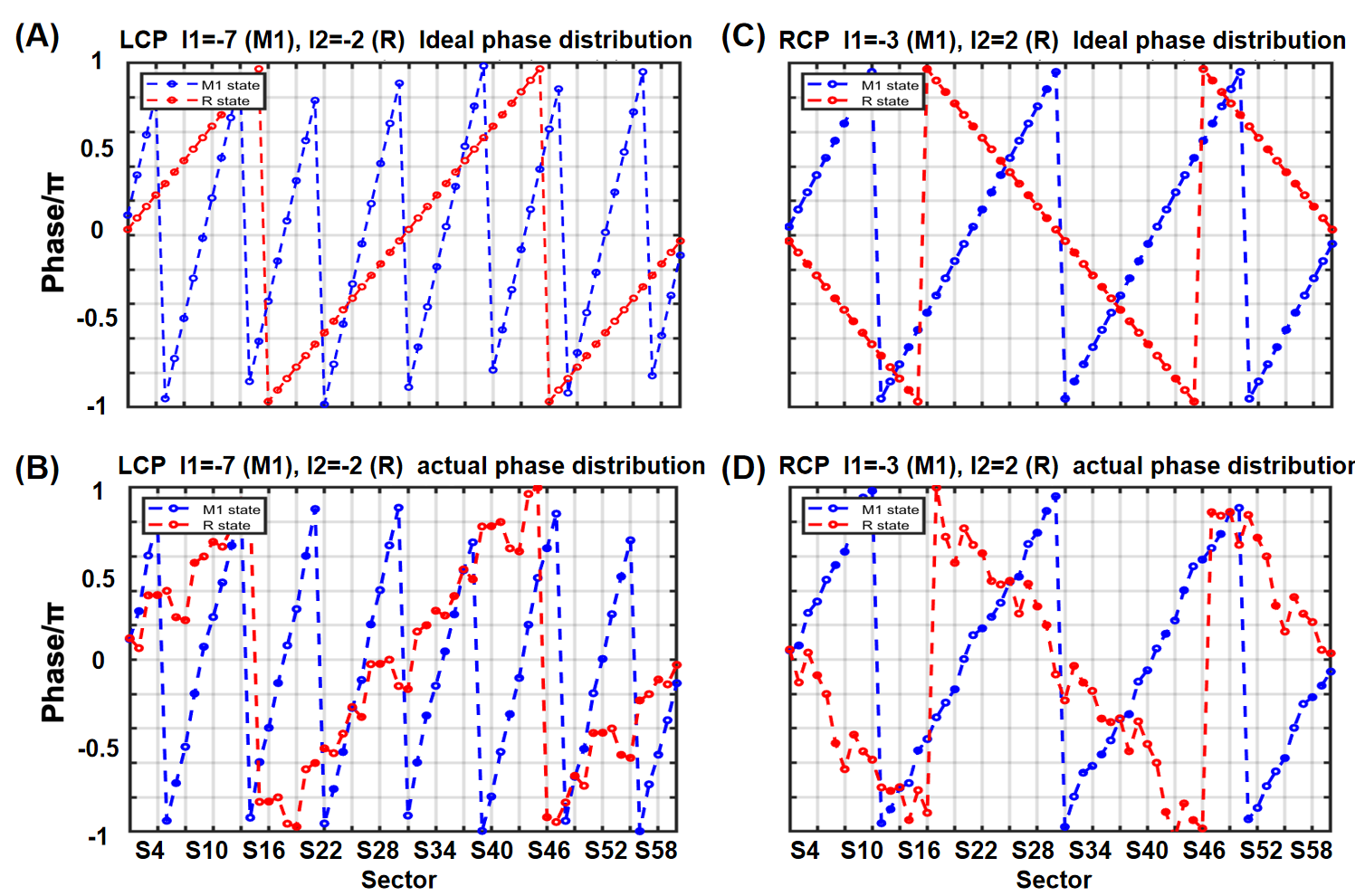


**Fig. S5. Ideal and actual phase distributions for topological charge switching under LCP and RCP incidence.** (A) Ideal phase distribution for topological charge switching from -7 (M1) to -2 (R) as a function of sector number under LCP incidence. (B) Actual phase distribution of the selected meta-atom, showing close agreement with the ideal distribution in (A). (C) Ideal phase distribution for topological charge switching from -3 (M1) to 2 (R) as a function of sector number under RCP incidence. (D) Actual phase distribution of the selected meta-atom, showing close agreement with the ideal distribution in (C).

Our design framework in the main text naturally accommodates dual‑circular polarization. For a chosen set of topological charges ($l_{1}$,$l_{2}$,$l_{3}$,$l_{4}$) that satisfy condition described in the first equation in Eq. (5), we can obtain the required propagation phase difference and sum and the corresponding rotation angles. The algorithm then searches the meta‑atom library to find the best‑matching structures for each azimuthal sector. As shown in Fig. S5, this approach enables the design of metasurfaces that generate OAM modes with four distinctive topological charges by leveraging the two circular polarizations and the two-phase states of VO_2_. For instance, we have designed a metasurface that can switch between TC = -7 (M1) and TC = -2 (R) under LCP incidence, and between TC = -3 (M1) and TC = 2 (R) under RCP incidence, demonstrating flexible and large TC leaps at both circular polarizations.

For applications that require operation with unpolarized light, the metasurface would simultaneously generate the two OAM states (for LCP and RCP) corresponding to the chosen topological charges. The far‑field pattern will be the incoherent sum of these two vortex beams. If the two states have opposite topological charges (e.g., $l_{1}=-l_{3}$,$l_{2}=-l_{4}$), the combined pattern will lose a clear vortex signature. However, by appropriate choice of the four topological charges, one can still achieve distinct switching behaviors. For instance, the desired OAM mode can be filtered out using a quarter-wave plate followed by a linear polarizer.

1. **Potential Design Strategies for Polarization-Versatile Operation**

In addition to the above theory, there are other possible strategy to extend the active VO_2_ metasurface into polarization versatile platform.

**Polarization-multiplexed metasurfaces:** One could design a bilayer metasurface where the top layer independently modulates LCP and the bottom layer modulates RCP. Each layer would be optimized following the single-polarization design principle presented in our work. This approach decouples the two polarizations at the expense of increased fabrication complexity and thickness.

**Chiral meta-atoms:** Introducing intrinsic chirality into the meta-atom design could break the symmetry between LCP and RCP responses. By engineering chiral resonances, one might independently control the phase for the two circular polarizations, thereby relaxing the requirements of Eq. (5).

**Supplementary Note 6:The design and performance of -1 - 4 OAM mode switching**

To further demonstrate the flexibility of the proposed method, we applied the same procedure to construct another active metasurface whose topological charge could switch between -1 and 4. This new case, designed under the same principle, underscores the repeatability and generality of our method.


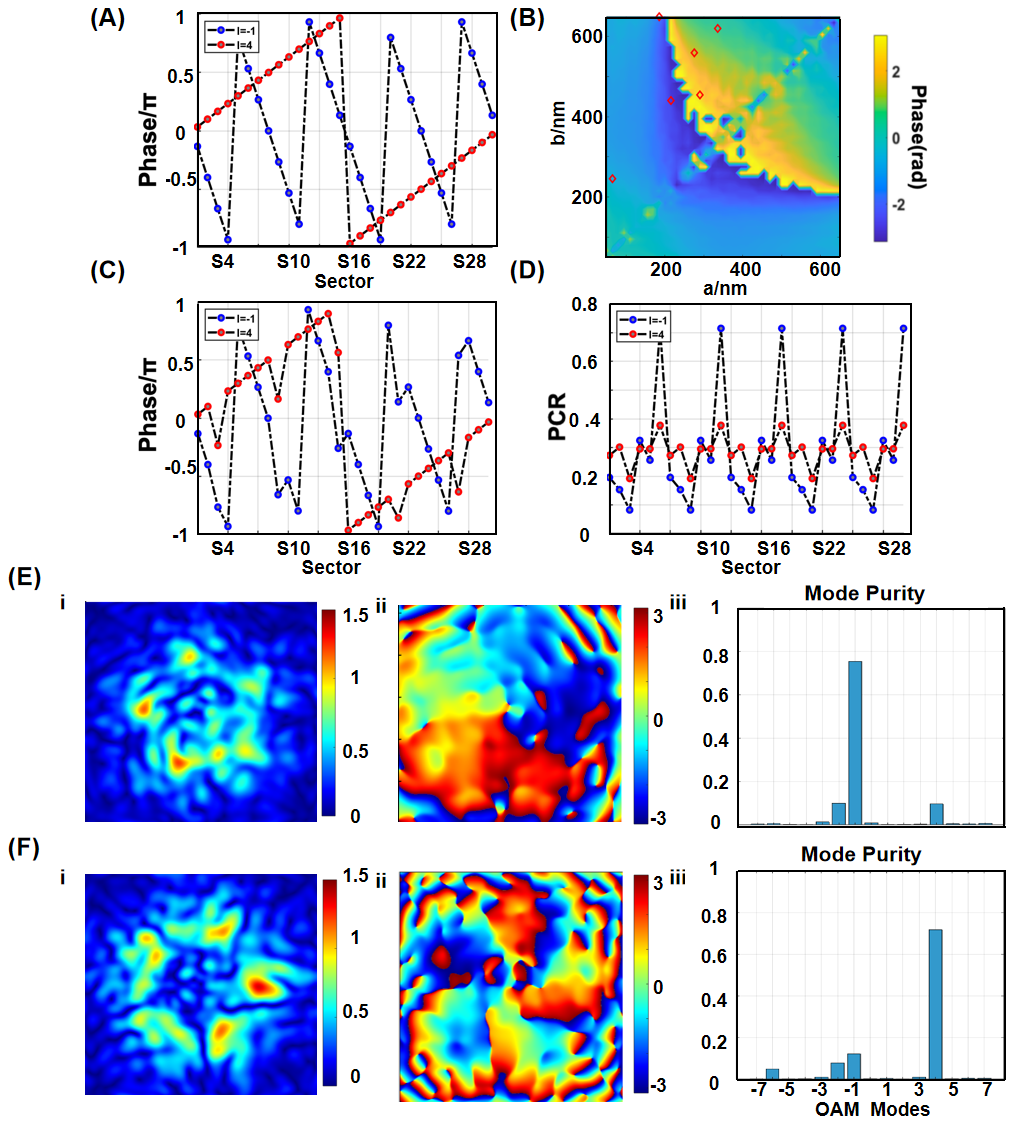


**Fig. S6. -1 - 4 OAM mode switching metasurface design and performance.** (A) Ideal phase distribution for topological charge switching from -1 to 4 as a function of sector number. (B) Selected structural parameters for the 30 sectors. Only six distinct meta-atom parameters are selected due to the periodic repetition of the phase difference constraint every six sectors. (C) Actual phase distribution of the fabricated metasurface, showing close agreement with the ideal distribution in (A). (D) PCR values for each sector in both dielectric (M1) and metallic (R) states. In the dielectric state, PCR fluctuates around 20% with a maximum reaching 70%, while in the metallic state, it mainly distributes around 30%. (E, F) Metasurface results showing (i) intensity distributions, (ii) phase distributions, and (iii) corresponding OAM mode purities for the target polarization states in the (E) M1 and (F) R phases. Both states exhibit mode purities exceeding 70%.

As shown above, Fig. S6 presents the design and simulation validation of an actively tunable metasurface for switching the orbital angular momentum (OAM) topological charge from -1 to 4, leveraging the phase transition of VO₂. Fig. S6(A) illustrates the ideal linear phase progression required across 30 azimuthal sectors to achieve this switch. Owing to the periodic recurrence of the phase-difference constraint every six sectors, only six distinct meta-atom geometries are selected to construct the entire device, as shown in Fig. S6(B). However, the rotation of each sector remains different (detailed in Table S3). The combination of propagation phase of the chosen meta-atoms with the corresponding PB phase response leads the final phase profile obtained in Fig. S6(C). It can be clearly seen that such result closely resembles the ideal phase design in Fig. S6(A), marking the success in meta-atom selection. Fig. S6(D) plots the PCR for each sector in both the M1 and R states of VO₂. In the M1 state, the PCR fluctuates near 20% with a maximum of 70% and the total reflection rate is 73.6%, while in the R state it is primarily distributed around 30%, with a total reflection rate of 71.2%. Finally, Fig. S6(E, F) display the output intensity profiles, phase distributions, and corresponding OAM mode spectra of the fabricated metasurface in the M1 and R states, respectively. The measured mode purities reach 75.5% for the TC = -1 output and 71.9% for the TC = 4 output, demonstrating high-fidelity mode generation and reliable switching. Collectively, these results underscore the robustness and repeatability of the design strategy for higher-order topological charge manipulation, paving a practical path towards OAM-based applications such as optical communications.


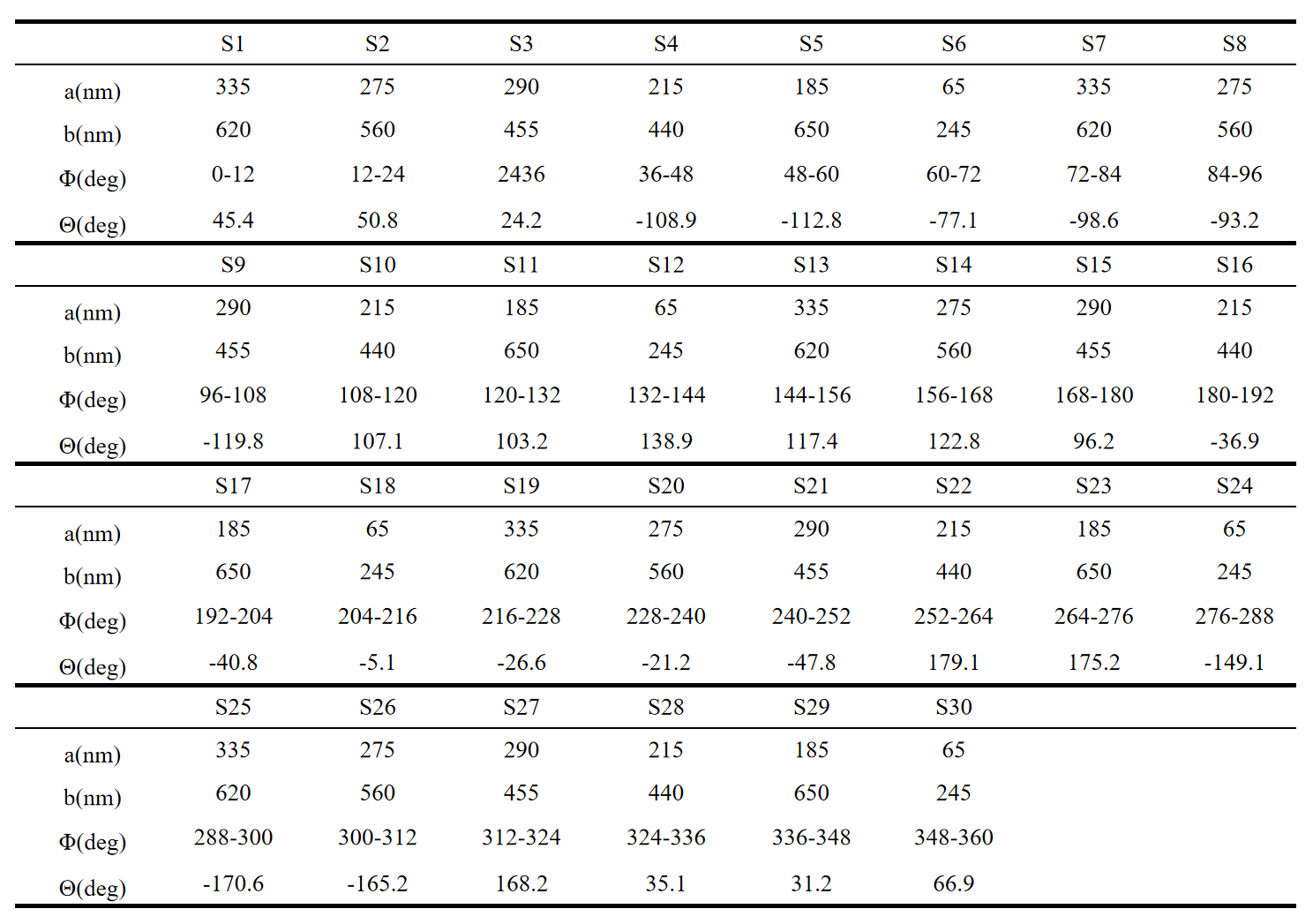
Table S3. The parameters of the meta-atoms for 4 - -1 metasurface.

**Reference**

1. Chen, Benwen, et al. "Programmable terahertz metamaterials with non-volatile memory." Laser & Photonics Reviews 16.4, 2100472, 2022.
2. Guo, Tingbiao, et al. "Durable and programmable ultrafast nanophotonic matrix of spectral pixels." Nature Nanotechnology 19.11, 1635-1643 (2024).
3. Houska, J., et al. "Characterization of thermochromic VO_2_ (prepared at 250°C) in a wide temperature range by spectroscopic ellipsometry." Applied Surface Science 421 : 529-534, 2017.
